# Post-irradiation cutaneous angiosarcoma

**DOI:** 10.1186/1757-1626-1-241

**Published:** 2008-10-16

**Authors:** Rohtesh S Mehta, Michael Mikhail

**Affiliations:** 1 Resident (PGY-3), Department of Internal Medicine, Mercy Catholic Medical Center/Drexel University School of Medicine, Mercy Fitzgerald Hospital, 1500 Lansdowne Avenue, Medical Science Building, Darby, PA, 19023, USA; 2 Consultant Physician, Department of Hematology and Oncology, Mercy Catholic Medical Center, Mercy Fitzgerald Hospital, 1500 Lansdowne Avenue, Medical Science Building, Darby, PA, 19023, USA

## Abstract

Angiosarcoma is a rare and highly malignant tumor with potential to recur despite treatment, and carries a poor prognosis. Previous radiation therapy and lymphedema are some of the known risk factors. We present a case of cutaneous angiosarcoma which occurred at lumpectomy site in a patient with a history of breast cancer and radiation to the breast. The tumor kept on recurring repetitively despite continual treatments, and the patient finally succumbed to the disease roughly four years after initial diagnosis.

## Introduction

The sources for our literature search included the data base PubMed (Medline) 1966-current, Ovid MEDLINE(R) 1950 to July Week 2 2008, and Cochrane Database of Systematic Reviews (EBM Reviews).

## Case presentation

This is a case of an unfortunate 85 year old Caucasian female with a past medical history significant for diabetes, hypertension, lung cancer status-post right middle lobe lobectomy in 1998, and left breast invasive cancer status-post lumpectomy and radiation therapy in 2000, who developed a cutaneous angiosarcoma at the site of lumpectomy scar in 2004. She underwent left mastectomy with subsequent treated with paclitaxel (10/5/04 to 1/10/05). She remained in remission since then, but with residual chemotherapy related peripheral neuropathies. During one of the routine out-patient oncology clinic follow-up visits on 1/23/07, she was found to have erythema of the medial third of the mastectomy scar along with one inch dark purplish lesion which was fixed to the underlying structures. Computed axial tomography (CAT) scans of the chest, abdomen and pelvis did not reveal any evidence of tumor. Owing to the attachment of the lesion to the chest wall and indurated skin around the lesion, she was not deemed a suitable candidate for surgical resection. Consequently, she received albumin-bound paclitaxel chemotherapy from 2/6/07 to 5/8/07 with a remarkable visible response. She had a complete resolution of the visible tumor from her anterior chest wall with no erythema or palpable masses. Afterwards, in 08/07 she underwent wide surgical excision of left breast mastectomy scar with skin grafting and flap reconstruction of chest wall by mobilization of the right breast towards the left. The pathology report of the specimen revealed recurrent high grade multi-focal angiosarcoma with positive deep margins, but no lymphovascular invasion. Re-staging diagnostic studies of chest, abdomen and pelvis remained unchanged with no evidence of tumor masses. On a follow-up visit in 11/07, not surprisingly, she was noted to have lesions at the scar site along with right sided breast nodules highly suspicious of the recurrent disease, but she refused any surgical interventions. She was started on chemotherapy with 3 weeks on and 1 week off cycles of Gemcitabine for a total of 12 treatments. She tolerated the therapy well but for residual toxicities such as fatigue and neutropenia which was controlled with Filgrastim. She once again showed striking improvement with complete resolution of all visible lesions and no palpable nodules. She continued to be in complete remission for 2 months post chemotherapy. She was feeling reasonable fine until July 2008 when she presented to the emergency department with worsening shortness of breath. Chest X-ray demonstrated a large left sided pleural effusion; she underwent thoracentesis and the fluid cytology was highly suspicious for malignant cells. She refused any further management and opted for comfort care. She finally passed away peacefully in July 2008 – almost 4 years after the initial diagnosis of angiosarcoma.

## Discussion

Angiosarcoma is an uncommon but aggressive malignancy arising from the vascular endothelial cells. About 1% of all adult cancers are comprised of sarcomas, while 2% of all soft tissue sarcomas are comprised of angiosarcomas [1-3]. They are notorious for local recurrences, systemic metastases, challenging treatment and an overall poor prognosis. These are categorized as (1) cutaneous angiosarcoma (2) angiosarcoma of deep soft tissues (3) angiosarcoma of bone, and (4) breast angiosarcoma. Cutaneous angiosarcoma has several variants such as (a) angiosarcoma of the scalp and face, (b) lymphedema-associated angiosarcoma, such as after mastectomy (Stewart-Treves syndrome), (c) radiation-associated angiosarcoma, and (d) epithelioid angiosarcoma [4]. Most of the cutaneous angiosarcomas occur in the head and neck region.[2] The risk of radiation induced sarcomas have been estimated to range from 0.03 to 0.8%, with radiadiotherapy doses ranging from 3000 to 12,440 cGy and a latency period of up to 12 years for the development of sarcoma from initial radiation therapy [5,6]. About 1/3^rd ^of all metastatic angiosarcomas occur in previously irradiated field [7].

In a study of 82 patients with angiosarcoma [2] the mean age of diagnosis was 65 years, with a range of 22 to 91 years, and 44% were noted in females, and 11% occurred in the setting of lymphedema or previous radiation. With regards to the location, 40% were found in skin, 27% in deep soft tissue, 10% in bone and 9% in breast. Another retrospective study [8] determined the median age of 52 at the time of diagnosis, with majority (65%) occurring in females. The most common location was found to be breast (38%), skin (21%) and superficial soft tissues (13%). Metastasis at the time of diagnosis was found in 27% of the patients.

Cutaneous angiosarcoma initiates as a "bruise-like" patches on the skin, and later progress to become violaceous with ill-defined nodular appearance. Stewart-Treves syndrome (angiosarcoma arising in the setting of in chronic lymphedema) and radiation-associated angiosarcoma usually start as violaceous infiltrating plaques or nodules. Because of similar physical findings, these lesions should be distinguished from hemangiomas or "simple bruises" [4]. Some of the other symptoms include pain, swelling, or bleeding. Histologically, these tumors can have various patterns such as vascular channels, sheets of cells, cells of undifferentiated morphology or a mixed picture [1]. Immunohistochemical staining with Factor VIII and UEA-I (Ulex europaeus agglutinin I) is usually positive in tumor cells of vascular differentiation, the latter being reported as more sensitive, but less specific for angiosarcomas [1].

Treatment consists of wide-margin excision whenever feasible, otherwise palliative chemotherapy or radiotherapy should be considered. Surgery is used as the first-line treatment in a majority (80%), followed by chemotherapy and radiotherapy in 38 and 30% of patients respectively [8]. Some of the chemotherapeutic agents used in angiosarcoma include paclitaxel, and doxorubicin with or without interferon-α2a or ifosfamide, and gemcitabine. A retrospective multicenter study conducted by Schlemmer et al [9] demonstrated the efficacy of Paclitaxel in angiosarcoma. 13 out of 32 patients in the study had received doxorubicin, 5 in combination with ifosfamide as 1^st ^line and 3 had received ifosfamide as 2^nd ^line chemotherapy prior to treatment with paclitaxel. Complete remission (CR) was observed in one, partial response (PR) in 19, with an overall response rate of 62.5%. In patients with angiosarcoma of face and scalp, the overall response rate was 75% (1 CR and 5 PR), while it was 58% (14 PR) in patients with angiosarcoma of other sites. The progression free survival was 7.6 months for all patients. A phase II prospective study [7] of metastatic angiosarcoma patients evaluated the efficacy of weekly Paclitaxel. 22 of 30 patients had previous surgery and 14 had previous anthracyclin based chemotherapy. 15, 11, 6 and 4 patients respectively received 3, 4, 5 and 6 cycles of paclitaxel, out of which 1 patient had grade 4 toxicity (neutropenia), 27 patients had grade 2 and 3 toxicities including myalgias (15/27), leuco-neutropenia (14/27), infections (6/27), and anemia (4/27). Of 23 patients available for the final analysis, 4 (14%) had partial response, 14 (56%) had stable disease and 7 (30%) had progressive disease, with an overall tumor-control rate of 70% (stable disease + partial response). Retrospective studies indicate that although Paclitaxel is efficacious in the treatment of angiosarcoma, it may not be superior to non-taxane base chemotherapies [10]. Individual reports indicate favorable results with combination therapy using liposomal doxorubicin and interferon-α2a. A complete response for more than 15 months was maintained in a patient with angiosarcoma of forehead and multiple bilateral pulmonary nodules [11] Gemcitabine has also been determined to efficacious in isolated reports [12].

Angiosarcomas have a poor prognosis even with treatment. Studies [8] demonstrated a relapse rate of 56% and death in 47% of patients within a median follow-up period of 1.8 years. 5-year survival is about 23% and 48% respectively in patients with and without metastasis at the time of diagnosis [8]. Bone and liver metastases are considered to be significant adverse prognostic factors [8]. Median survival ranges from 21 to 40 months depending upon the study [8,10].

Given the vascular origin of the tumor, it may not be surprising to potentially discover a role of some of the novel angiogenesis inhibitors in the treatment this tumor. Prospective trials evaluating various treatment options are limited owing to the rarity of this tumor. Further studies need to be conducted to truly unravel this enigmatic entity.

## Consent statement

Consent could not be obtained as the the next of kin could not be found.

## Competing interests

The authors declare that they have no competing interests.

## Authors' contributions

RSM did the literature search, compiled the patient's records and composed the paper. MM provided invaluable suggestions on writing the paper, in addition to providing the comprehensive patient data. Both of the authors read and approved the final manuscript.
